# Supplementary material for: Hypermethylation of SCAND3 and Myo1g Gene Are Potential Diagnostic Biomarkers for Hepatocellular Carcinoma
Source: Cancers (Basel). 2020 Aug 18;12(8):2332. doi: 10.3390/cancers12082332 (PMC7465898; doi:10.3390/cancers12082332)
Supplement: Supplementary file 1 [file cancers-12-02332-s001.pdf]

*Supplemental material*

# Hypermethylation of SCAND3 and Myo1g Gene Are Potential Diagnostic Biomarkers for Hepatocellular Carcinoma

**Fei Xu, Lulu Zhang, Yuxia Xu, Di Song, Wenting He, Xiaomeng Ji and Jianyong Shao**

**Table S1.** Clinicopathological characteristics of the 20 HCC patients.

| Characteristics | Values |
|---|---|
| **Sex** | |
| Male | 16 |
| Female | 4 |
| **Age (years)** | 53.16 ± 4.17 |
| **Tumor number** | |
| Single | 15 |
| Multiple | 5 |
| **BCLC stage** | |
| 0 | 8 |
| A | 7 |
| B | 5 |
| C | 0 |
| **HBV** | |
| Positive | 14 |
| Negative | 6 |
| **Liver cirrhosis** | |
| Positive | 7 |
| Negative | 13 |

*Methylation Data Analysis*

For digital droplet PCR (ddPCR), after PCR amplification, the 96-well PCR plates were placed into the Bio-Rad QX200 droplet reader and data were analyzed using QuantaSoft software (Bio-rad). The absolute quantification (ABS) experiment design was used in the data analysis. The number of positive and negative droplets in each sample was counted by QuantaSoft software. In our present study, the positive droplets (P) were regarded as a targeted gene (SCAND3 or Myo1g) methylated, the negative droplets (N) were regarded as reference gene GAPDH. The ratio between the number of P and the total number of P + N was used to represent the absolute methylation level for each sample. In other word, the percentage of methylation for each sample was calculated as % methylation = 100 × [P / (N + P)].

For methylation-specific PCR (MSP), the optimal cutoff value of CT (cycle threshold) in MSP assay for SCAND3 and Myo1g methylation was established based on the receiver operating characteristics curve (ROC) analysis [1-3]. All tested samples were categorized as methylated or unmethylated based on the cutoff values. The optimal cutoff of CT value for SCAND3 was 20. If CT value was < 20 cycles, a sample was considered as SCAND3 methylated; if CT value was ≥ 20 or not measurable, a sample was considered as SCAND3 unmethylated. The optimal cutoff of CT value for Myo1g was 23. If CT value was < 23 cycles, a sample was considered as Myo1g methylated; if CT value was ≥ 23 or not measurable, a sample was considered as Myo1g unmethylated. The test result of each sample was acceptable only when the CT value of the reference gene GAPDH was ≤ 30.

## References

1. Ang, P. W.; Toh, H. B.; Iacopetta, B.; Soong, R., An improved quality control for bisulfite-PCR-based DNA

methylation analysis: cycle threshold value. *Clinical chemistry and laboratory medicine* **2008,** *46*, 1117–1721.
2. Oh, T. J.; Oh, H. I.; Seo, Y. Y.; Jeong, D.; Kim, C.; Kang, H. W.; Han, Y. D.; Chung, H. C.; Kim, N. K.; An, S., Feasibility of quantifying SDC2 methylation in stool DNA for early detection of colorectal cancer. *Clinical Epigenetics* **2017,** *9*, 126.
3. Shan, M.; Yin, H.; Li, J.; Li, X.; Wang, D.; Su, Y.; Niu, M.; Zhong, Z.; Wang, J.; Zhang, X.; Kang, W.; Pang, D., Detection of aberrant methylation of a six-gene panel in serum DNA for diagnosis of breast cancer. *Oncotarget* **2016,** *7*, 18485–18494.

© 2020 by the authors. Licensee MDPI, Basel, Switzerland. This article is an open access article distributed under the terms and conditions of the Creative Commons Attribution (CC BY) license (http://creativecommons.org/licenses/by/4.0/).
